# Longitudinal surface measurements of human blastocysts show that the dynamics of blastocoel expansion are associated with fertilization method and ongoing pregnancy

**DOI:** 10.1186/s12958-022-00917-2

**Published:** 2022-03-19

**Authors:** Eva S. van Marion, Effrosyni A. Chavli, Joop S. E. Laven, Régine P. M. Steegers-Theunissen, Maria P. H. Koster, Esther B. Baart

**Affiliations:** 1 Division of Reproductive Endocrinology and Infertility, Department of Obstetrics and Gynaecology, Erasmus MC, University Medical Center, PO Box 2040, 3000 CA Rotterdam, The Netherlands; 2 Department of Obstetrics and Gynaecology, Erasmus MC, University Medical Center, Rotterdam, the Netherlands; 3 Department of Developmental Biology, Erasmus MC, University Medical Center, Rotterdam, The Netherlands

**Keywords:** Time-Lapse Imaging, Blastocyst, Embryonic Development, Pregnancy Outcome, Intracytoplasmic Sperm Injections

## Abstract

**Background:**

Despite all research efforts during this era of novel time-lapse morphokinetic parameters, a morphological grading system is still routinely being used for embryo selection at the blastocyst stage. The blastocyst expansion grade, as evaluated during morphological assessment, is associated with clinical pregnancy. However, this assessment is performed without taking the dynamics of blastocoel expansion into account. Here, we studied the dynamics of blastocoel expansion by comparing longitudinal blastocoel surface measurements using time-lapse embryo culture. Our aim was to first assess if this is impacted by fertilization method and second, to study if an association exists between these measurement and ongoing pregnancy.

**Methods:**

This was a retrospective cohort study including 225 couples undergoing 225 cycles of in vitro fertilization (IVF) treatment with time-lapse embryo culture. The fertilization method was either conventional IVF, intracytoplasmic sperm injection (ICSI) with ejaculated sperm or ICSI with sperm derived from testicular sperm extraction (TESE-ICSI). This resulted in 289 IVF embryos, 218 ICSI embryos and 259 TESE-ICSI embryos that reached at least the full blastocyst stage. Blastocoel surface measurements were performed on time-lapse images every hour, starting from full blastocyst formation (tB). Linear mixed model analysis was performed to study the association between blastocoel expansion, the calculated expansion rate (µm^2^/hour) and both fertilization method and ongoing pregnancy.

**Results:**

The blastocoel of both ICSI embryos and TESE-ICSI embryos was significantly smaller than the blastocoel of IVF embryos (beta -1121.6 µm^2^; 95% CI: -1606.1 to -637.1, beta -646.8 µm^2^; 95% CI: -1118.7 to 174.8, respectively). Still, the blastocoel of transferred embryos resulting in an ongoing pregnancy was significantly larger (beta 795.4 µm^2^; 95% CI: 15.4 to 1575.4) and expanded significantly faster (beta 100.9 µm^2^/hour; 95% CI: 5.7 to 196.2) than the blastocoel of transferred embryos that did not, regardless of the fertilization method.

**Conclusion:**

Longitudinal blastocyst surface measurements and expansion rates are promising non-invasive quantitative markers that can aid embryo selection for transfer and cryopreservation.

**Trial registration:**

Our study is a retrospective observational study, therefore trial registration is not applicable.

**Supplementary Information:**

The online version contains supplementary material available at 10.1186/s12958-022-00917-2.

## Background

Selecting the most viable embryo for transfer is a daily challenge for in vitro fertilization (IVF) clinics. The golden standard for embryo selection remains morphological assessment at different developmental stages. Despite significant refinements made in this assessment, the transfer of embryos with optimal morphological characteristics can still lead to implantation failure and pregnancy loss [1]. In addition, the preference for single embryo transfer (SET) makes an improved embryo selection method even more important. Therefore, much research effort in the field of IVF remains dedicated to new approaches to improve embryo selection.

Extended culture of embryos up to five or six days post-fertilization and the selection of embryos that are able to reach the blastocyst stage is one of the approaches to improve embryo selection. After the cleavage divisions, the embryo undergoes compaction and then the first lineage specification results in the formation of the blastocyst, comprising of an outer layer of polarized epithelial cells, the trophectoderm (TE), a compact inner cell mass (ICM), and a fluid-filled cavity, the blastocoel. A sufficient amount of functional TE cells is required for proper blastocyst expansion as they accumulate ions in the blastocoel. This causes an osmotic gradient, which promotes fluid inflow into the blastocoel across the TE cells, driving blastocoel expansion [2]. Clinical trials have suggested that blastocyst transfers achieve higher implantation and live birth rates compared to cleavage stage embryos [3, 4]. From a biological point of view, embryo culture until blastocyst formation functions as a filter for the selection of embryos with the highest developmental potential [5–7]. During the first cleavage divisions the embryo uses maternal RNA transcripts that are gradually degraded while the embryonic genome is gradually activated [8, 9]. Further culture until the blastocyst stage allows embryo assessment after embryonic genome activation and morphological examination of both embryonic lineages (TE and ICM) [10]. Furthermore, embryos with complex aberrant chromosomal constitutions are more likely to arrest at the cleavage stages [11]. However, embryos with chromosomal abnormalities can still reach the blastocyst stage, therefore invasive techniques that involve embryo biopsy and genetic screening are often incorporated in order to identify chromosomally normal embryos for transfer [12].

Despite all research efforts during this era of time-lapse embryo culture and novel time-lapse morphokinetic (TLM) parameters, a morphological grading system is still routinely being used for embryo selection at the blastocyst stage. The most widely used morphological grading system, based on Gardner and Schoolcraft, scores the degree of blastocoel expansion and hatching status, size and compactness of the ICM and the cohesiveness and number of TE cells [10, 13]. It is shown that the blastocyst expansion grade is associated with clinical pregnancy [14–16]. However, evaluation according to this grading system remains subjective and morphological assessment is performed on a specific time point without taking the dynamics of blastocoel expansion into account [17].

A more quantitative approach would be to repeatedly measure the actual size of the blastocoel by using time-lapse embryo culture. However, it is unclear whether an association exists between blastocoel size over time and embryo implantation. One previous study compared the cross-sectional area of blastocysts that did or did not result in implantation and they found no significant difference in the slope of the averaged expansion curves [18]. These results were based on a small cohort of 53 implanted blastocysts and 15 non-implanted blastocysts. Also, analysis using a regression slope does not correct for the fact that embryos originating from the same couple show a comparable developmental pattern, a phenomenon called clustering [19]. In addition, fertilization methods and sperm origin have been observed to impact on time-lapse morphokinetics of preimplantation embryos [19–22]. Thus, to understand if measuring blastocoel expansion could aid in embryo selection, it is necessary to investigate the impact of these factors. Therefore, our aim was to first investigate whether an association exists between the dynamics of blastocoel expansion and fertilization method, and second if this can be indicative of implantation and ongoing pregnancy.

## Materials and methods

### Study design and participants

**Fig. 1:**
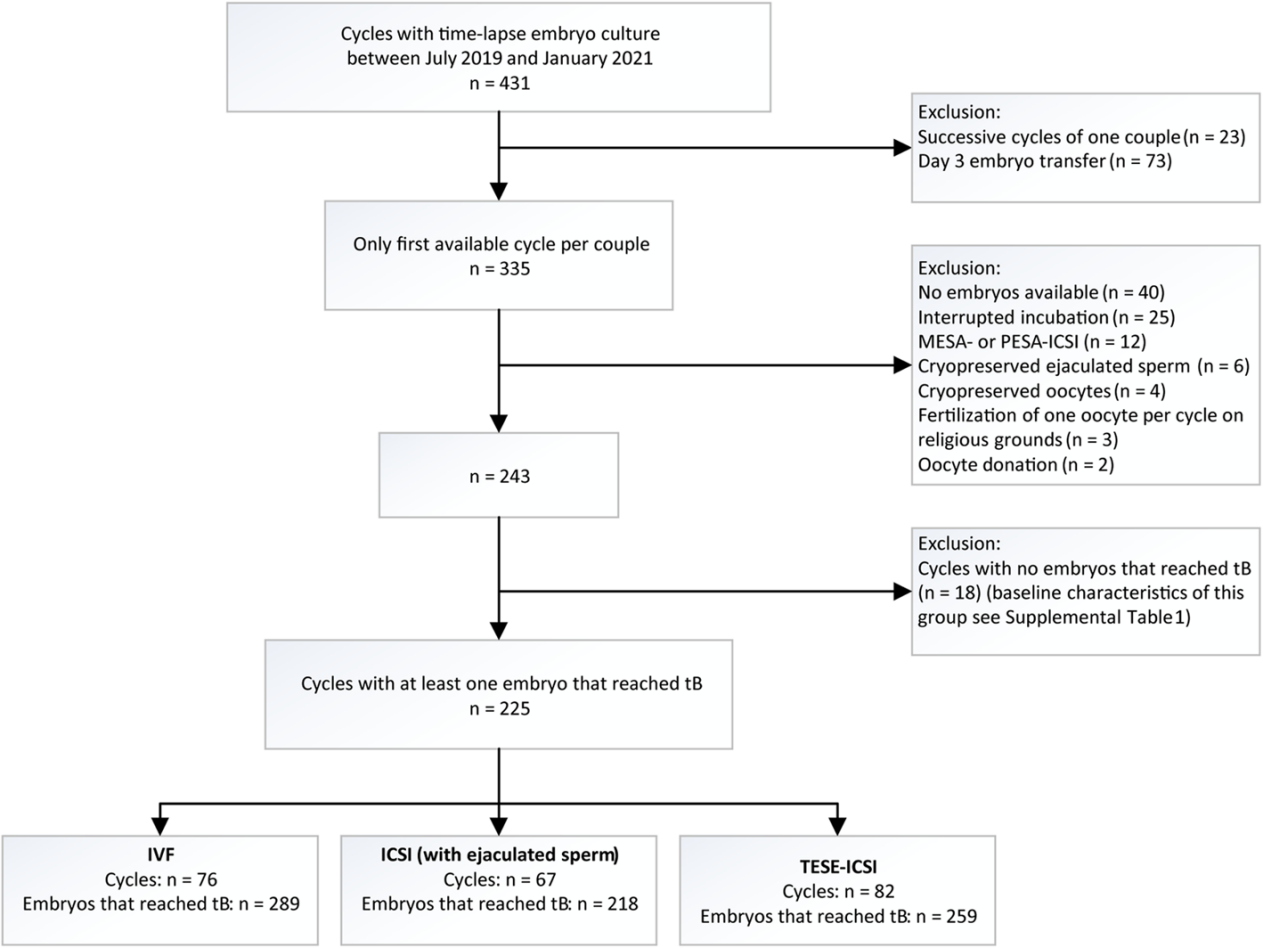
Flowchart of included and excluded cycles. Abbreviations: tB, time to full blastocyst; IVF, in vitro fertilization; ICSI, intracytoplasmic sperm injection; TESE-ICSI, testicular sperm extraction combined with intracytoplasmic sperm injection

This was a retrospective cohort study including couples that underwent an IVF treatment cycle at the Erasmus MC, University Medical Center Rotterdam, between July 2019 and January 2021. During this study period all patients with autologous, fresh oocytes that were fertilized using IVF or ICSI with either ejaculated sperm (ICSI group) or with surgically retrieved testicular sperm (TESE-ICSI group) that were cultured in a time-lapse incubator (EmbryoScope, Vitrolife, Göteborg, Sweden) were included. Patients are routinely referred by the treating physician for IVF or ICSI treatment based on the total motile sperm count observed during routine semen analysis. Our clinic annually performs around 250 IVF cycles, 350 ICSI cycles and 150 TESE-ICSI cycles. All TESE-ICSI cycles were assigned to time-lapse embryo culture, while this was based on space availability on the day before oocyte pick up for IVF and the other ICSI cycles. Cycles with both single- and double fresh embryo transfer (SET and DET) were included, as well as cycles without a fresh embryo transfer. From couples undergoing multiple cycles during the study period, only data from their first available treatment cycle were included. Cycles resulting in day 3 embryo transfer or without time-lapse data of at least one embryo that reached the full blastocyst stage were also excluded. Moreover, cycles with cryopreserved ejaculated sperm and cycles in which part of the oocytes were frozen on religious grounds, were excluded (Fig. 1).

### Ovarian stimulation, oocyte collection, oocyte insemination and injection

Women underwent routine ovarian stimulation by either a gonadotropin-releasing hormone (GnRH) -agonist or -antagonist co-treatment protocol with recombinant follicle stimulating hormone (Bemfola, Gedeon Richter, Belgium; or Gonal-F, Merck Serono, Switzerland; or Rekovelle®, Ferring, St. Prex, Switzerland) or highly purified urinary FSH (Menopur, Ferring, St. Prex, Switzerland) [23]. Ovarian stimulation protocols are standardized at our center and the distribution of GnRH-agonist or –antagonist protocols reflects policy changes for different fertilization methods over time and not patient selection. Human recombinant chorionic gonadotropin (hCG) (Ovitrelle®, Merck Serono, Switzerland, Pregnyl®, Organon, the Netherlands) was used as a trigger of final follicular maturation. After oocyte retrieval, oocytes were placed in fertilization medium (G-IVF, Vitrolife). Ejaculated sperm was washed in a commercially available discontinuous two layer (45%–90%) density gradient (SpermGrad, Vitrolife). On the day of oocyte pick up, the total motile count is routinely determined after sperm processing by gradient density centrifugation. Fertilization is always performed by ICSI should after sperm processing a total motile sperm count of < 3.10^5^ be recovered Where applicable, testicular sperm was retrieved and frozen, followed by thawing on the day of ICSI treatment as previously described [22]. Oocytes were fertilized according to routine IVF, ICSI with ejaculated sperm or TESE-ICSI procedures as described previously [22].

### Embryo culture, selection and transfer

Fertilized oocytes were placed in EmbryoSlide culture dishes (Vitrolife) and were cultured in an EmbryoScope time-lapse incubator (Vitrolife). The culture medium used was SAGE 1-step (Origio/Cooper Surgical, Trumbull, CT, USA) between July 2019 and December 2019 and Vitrolife G-TL (Vitrolife, Göteborg, Sweden) between December 2019 and January 2021. Embryos were cultured at 36.8 degrees Celsius, 7% O_2_ and 5% or 6% CO_2_ for SAGE 1-step or G-TL, respectively. Embryo selection for transfer was performed 112–116 h after fertilization by morphological assessment [10, 22]. Embryo selection was performed without taking time-lapse parameters into account. None of the embryos were subjected to assisted hatching or preimplantation genetic testing. Single or double embryo transfer was performed in the afternoon. Biochemical pregnancy was investigated 10 days after transfer by a urinary β-hCG test, and ongoing pregnancy was confirmed by a fetal heartbeat during an ultrasound at 12 weeks of gestation.

### Time-lapse imaging and blastocyst surface measurements

**Fig. 2:**
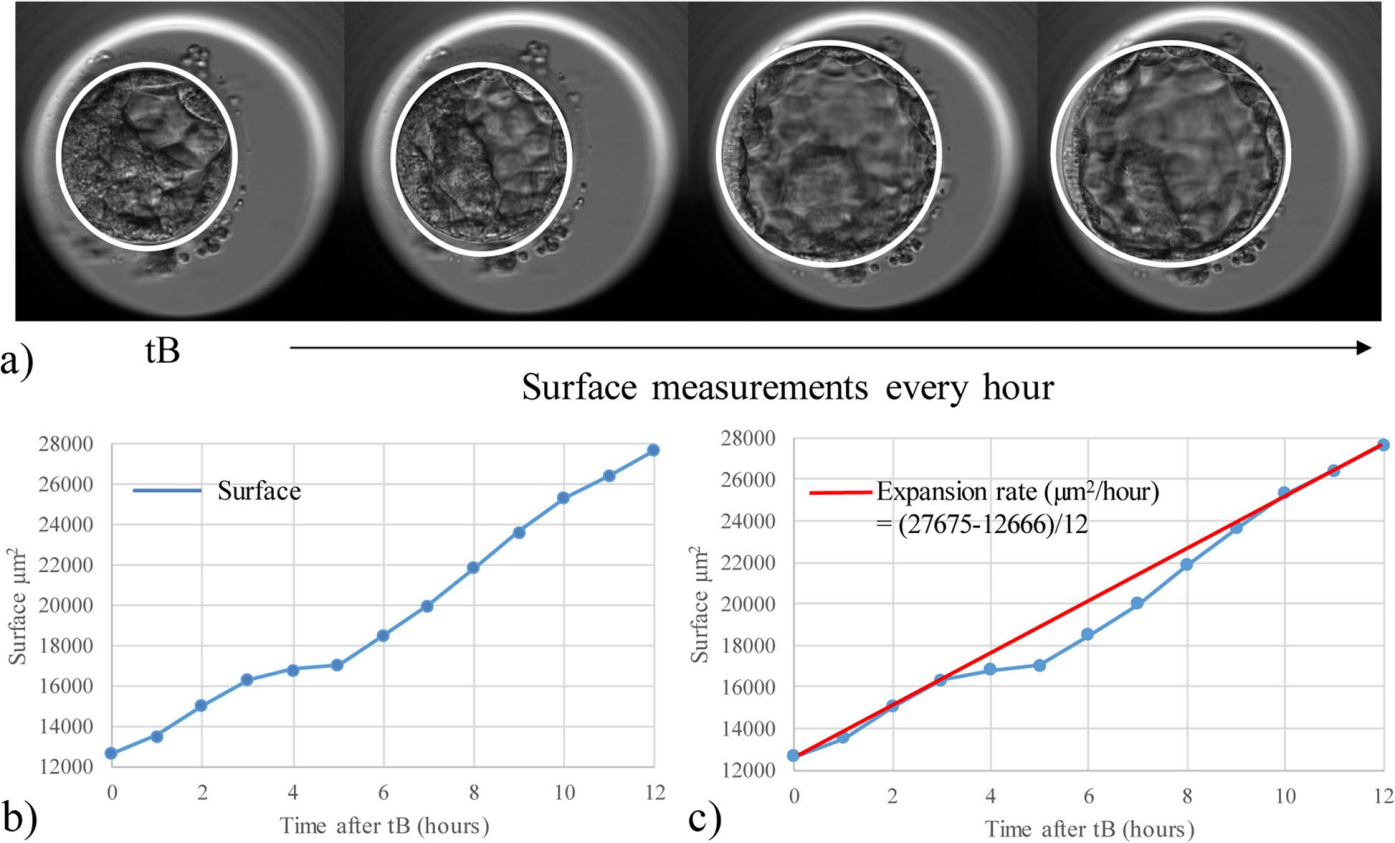
**a** Surface measurements were performed every hour by using the ellipse tool of the EmbryoViewer software, starting at tB. **b** Example of a blastocyst expansion curve. **c** Example of the calculated expansion rate (µm^2^/hour): maximum measured surface subtracted by the surface at tB, divided by the number of measurements in between. Abbreviations: tB, full blastocyst formation

The EmbryoScope (Vitrolife) records images automatically in seven focal planes every 10 min. The timing of full blastocyst formation (tB) was annotated as the last frame before zona pellucida thinning, according to published consensus definitions and guidelines [24]. The surface of the blastocyst was measured every hour in square micrometers, by using the ellipse tool of the EmbryoViewer software (Vitrolife). The ellipse was formed around the outer edge of the trophectoderm. The zona pellucida was not included in these measurements (Fig. 2a). Measurements were performed at the focus plane that contained the largest surface area. The first measurement was always performed on the first image where tB was reached. Fresh transferred embryos were measured every hour until selection for embryo transfer at 112–116 h post insemination or injection. Cryopreserved embryos were measured every hour until 116 h post insemination or injection. In some embryos, the first signs of hatching were observed as evidenced by herniation of one or a few TE cells. In these cases, measurements were continued as the expansion was observed to continue in all cases. All measurements were performed by the same investigator, blinded for the outcome of the fresh embryo transfer. From these measurements, the expansion rate was calculated for each embryo. This was done by first determining the time point where the blastocyst reached the largest surface area. The surface at tB was then subtracted from this maximum surface area, and this was divided by the number of measurements in between (in other words: the number of hours from tB; Fig. 2b, c).

### Statistical analysis

Baseline characteristics and treatment outcomes of all included cycles were tested for the assumption of normality. Because data were not normally distributed, a Kruskal–Wallis test was performed to compare data between different groups. Estimates are reported as medians and interquartile range (IQR). Categorical data were analysed with the Chi-square test/Fisher exact test. Two separate analyses were performed, one to analyse the association between blastocoel expansion and fertilization method and the second for blastocoel expansion and pregnancy outcome.

To analyse the association between fertilization method and blastocyst expansion, all transferred and cryopreserved embryos that reached at least tB were included. From these embryos, the surface was measured every hour starting at tB. These longitudinal surface measurements were used to investigate differences in blastocoel size over time between the three different fertilization methods (IVF, ICSI with ejaculated sperm and TESE-ICSI). For this purpose, a linear mixed model analysis was performed, using the IVF group as a reference group. This model compared differences in size of the blastocoel between the fertilization methods over the entire expansion trajectory. Each embryo follows its own developmental pattern resulting in a different time point for full blastocyst formation (tB). Therefore, we adjusted our model for tB. Next, we wanted to investigate potential differences in the pace of blastocoel expansion between the three fertilization method groups. Therefore, another linear mixed model analysis was performed to compare the calculated expansion rate between the three fertilization method groups.

To analyse the association between ongoing pregnancy and blastocyst expansion, fresh transferred embryos that reached at least tB were included. All fresh SETs were included and DETs resulting in a twin pregnancy or those that did not result in a pregnancy. From these embryos, the surface was measured every hour starting at tB. These longitudinal surface measurements were used to investigate differences in blastocoel size over time between the group that resulted in an ongoing pregnancy and those that did not. This was investigated by a linear mixed model analysis, using the group without an ongoing pregnancy as a reference group. This model was also adjusted for tB. A second model was additionally adjusted for female age because this is a known confounder of ongoing pregnancy. Next, we wanted to investigate potential differences in the pace of blastocoel expansion between the group that resulted in an ongoing pregnancy and those that did not. Therefore, another linear mixed model analysis was performed to compare the calculated expansion rate between embryos that resulted in a pregnancy and embryos that did not. This first model was crude, and the second model was adjusted for female age.

All linear mixed model analyses took clustering of embryos originating from one couple into account [19]. The models also correct for differences in the number of measurements between embryos, as this varies according to the time frame between tB and selection for transfer or cryopreservation. All statistical analyses were performed in the statistical package for the social sciences (SPSS), version 25. Two-sided *p*-values < 0.05 were considered statistically significant.

## Results

### Patient characteristics and treatment outcome

A total of 243 cycles from unique patient couples were available for analysis. From these, 18 cycles were excluded as no blastocysts were formed. For these excluded cycles, the baseline characteristics and treatment outcomes are shown in a table (Additional file 1). From the remaining 225 cycles, 76 involved IVF treatment, 67 ICSI treatment with ejaculated sperm and 82 ICSI treatment with testicular sperm. This resulted in 289 IVF embryos, 218 ICSI with ejaculated sperm embryos and 259 TESE-ICSI embryos that reached at least the full blastocyst stage (Fig. 1). Baseline characteristics and treatment outcomes of the included cycles are shown for the different fertilization methods (Table 1).

**Table 1:** Baseline characteristics and treatment outcomes of the included cycles

	**IVF** (*n* = 76)	**ICSI (ejaculated sperm)** (*n* = 67)	**TESE-ICSI** (*n* = 82)	***p*-value**
**Total number of embryos**				0.584
Transfer	76	59	78	
Freeze	213	159	181	
**Female age** (years)	35.2 (32.0–38.7)	33.4 (29.5–35.7)	33.2 (29.8–37.3)	0.020
**Male age** (years)	35.5 (32.0–39.0)	35.0 (30.0–38.0)	36.0 (32.0–42.3)	0.540
**Oocytes aspirated**	10 (6–12)	10 (7–14)	10 (7–13)	0.460
**Diagnosis**				< 0.001
Male factor	1 (1.3%)	34 (50.7%)	56 (68.3%)	
Female factor	47 (61.8%)	4 (6.0%)	0 (0%)	
Combined	2 (2.6%)	22 (32.8%)	26 (31.7%)	
Unexplained infertility	23 (30.3%)	7 (10.4%)	0 (0%)	
Other	3 (3.9%)	0 (0%)	0 (0%)	
**Stimulation Protocol**				< 0.001
GnRH-antagonist	47 (66.2%)	59 (92.2%)	45 (55.6%)	
GnRH-agonist	24 (33.8%)	5 (7.8%)	36 (44.4%)	
missing	5	3	1	
**Culture medium**				0.042
Sage1	17 (22.4%)	25 (37.3%)	33 (40.2%)	
Vitrolife G-TL	59 (77.6%)	42 (62.7%)	49 (59.8%)	
**Number of transferred embryos**				0.390
0	6 (7.9%)	10 (14.9%)	11 (13.4%)	
1	64 (84.2%)	55 (82.1%)	64 (78.0%)	
2	6 (7.9%)	2 (3.0%)	7 (8.5%)	
**Fertilization rate** (number of 2 PN/number of M2)				0.001
0–25%	3 (3.9%)	0 (0%)	4 (4.9%)	
25–50%	10 (13.2%)	8 (11.9%)	25 (30.5%)	
50–75%	27 (35.5%)	27 (40.3%)	34 (41.5%)	
75–100%	36 (47.4%)	32 (47.8%)	19 (23.2%)	
**Embryo usage rate** (number of transferred and cryopreserved embryos/number of 2 PN)				0.704
0–25%	5 (6.6%)	6 (9.0%)	6 (7.3%)	
25–50%	26 (34.2%)	30 (44.8%)	29 (35.4%)	
50–75%	23 (30.3%)	19 (28.4%)	28 (34.1%)	
75–100%	22 (28.9%)	12 (17.9%)	19 (23.2%)	
**Biochemical pregnancy**	42 (60.0%)	32 (56.1%)%)	29 (40.8%)	0.057
**Ongoing pregnancy**				0.767
Singleton	27 (38.6%)	21 (36.8%)	23 (32.4%)	
Twin	1 (1.4%)	1 (1.8%)	0 (0%)	

Each cycle is derived from a unique patient couple. Data are presented as number (%) or median (interquartile range). A *p*-value of < 0.05 was considered significant.

*Abbreviations:* *IVF* In vitro Fertilization, *ICSI* Intracytoplasmic Sperm Injection, *TESE-ICSI* Testicular Sperm Extraction combined with Intracytoplasmic Sperm Injection, *PN* Pronuclei, *M2* Metaphase 2

### Blastocoel size and expansion rate of IVF and ICSI embryos

**Fig. 3:**
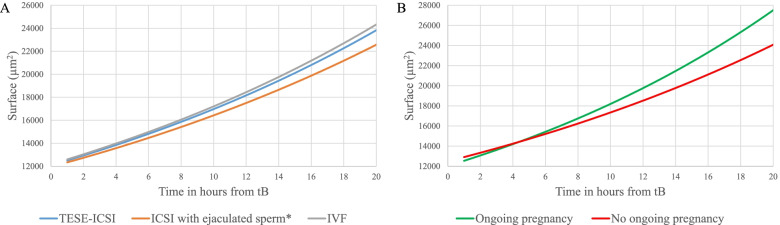
**a** Linear trend lines showing the blastocoel expansion from blastocysts originating from different fertilization methods (IVF; grey line [*n* = 289], ICSI with ejaculated sperm; orange line [*n* = 218], TESE-ICSI; blue line [*n* = 259]). **b** Linear trend lines representing blastocoel expansion of blastocysts resulting in no ongoing pregnancy (red line; *n* = 134) or ongoing pregnancy (green line; *n* = 69). *One embryo with early full blastocyst formation was excluded, because it was the only embryo with 43 measurements. Abbreviations: tB, time to full blastocyst; IVF, in vitro fertilization; ICSI, intracytoplasmic sperm injection; TESE-ICSI, testicular sperm extraction with intracytoplasmic sperm injection

Linear mixed model analysis showed that the blastocoel of ICSI embryos and TESE-ICSI embryos were significantly smaller than the blastocoel of IVF embryos at (beta -1121.6 µm^2^; 95% CI: -1606.1 to -637.1, beta -646.8 µm^2^; 95% CI: -1118.7 to 174.8, respectively) (Table 2, Model 1a). This means that the blastocoel size of ICSI embryos originating from ejaculated sperm was on average 1118.7 µm^2^ smaller during the entire expansion trajectory than IVF embryos. This is illustrated by blastocyst expansion trend lines of the three different fertilization methods (Fig. 3a).

**Table 2:** Linear mixed model analysis of blastocyst expansion surface measurements over time and the expansion rate, of all transferred and cryopreserved embryos compared between IVF, ICSI with ejaculated sperm and TESE-ICSI

	Model 1a Beta [95% CI] µm^2^				
	TESE-ICSI	*p*-value	ICSI (ejaculated sperm)	*p*-value	IVF
Surface	-646.8 [-1118.7 to 174.8]	0.007	-1121.6 [-1606.1 to -637.1]	< 0.001	ref
	Model 1b Beta [95% CI] µm^2^/hour				
Expansion rate	-43.7 [-113.5 to 26.1]	0.218	-93.2 [-165.0 to -21.0]	0.012	ref

Beta’s are reported as estimates in µm^2^ or µm^2^/hour. Model 1a: adjusted for tB; Model 1b: crude; A *p*-value of < 0.05 was considered significant.

*Abbreviations:* *tB* time to full Blastocyst, *IVF* In vitro Fertilization, *ICSI* Intracytoplasmic Sperm Injection, *TESE-ICSI* Testicular Sperm Extraction with Intracytoplasmic Sperm Injection, *ref* reference

Patients undergoing ICSI with ejaculated sperm were more often subjected to a GnRH-antagonist protocol than the IVF and TESE-ICSI patients (92.2% vs 66.2% vs 55.6%, respectively; *p*-value: < 0.001; Table 1). Also, resulting embryos of IVF treatment were less often cultured in Sage 1 culture medium than embryos originating from ICSI with ejaculated sperm and TESE-ICSI (22.4% vs 37.3% vs 40.2%, respectively; *p*-value: 0.042; Table 1). To exclude potential confounding factors, linear mixed model analysis was performed. This showed that blastocoel size was not different between the two stimulation protocols (beta -336.3 µm^2^; 95% CI: -784.6 to 112.1; Additional file 4). Blastocoel size was also found not to differ between the two culture media (beta 269.6 µm^2^; 95% CI: -159.2 to 698.4; Additional file 4).

Next, we compared the expansion rate of each embryo between the three groups. This analysis revealed that the blastocoel of ICSI embryos originating from ejaculated sperm expands significantly slower (beta -93.2 µm^2^/hour; 95% CI: -165.0 to -21.0) compared to the blastocoel of IVF embryos (Table 2, Model 1b). The expansion rate of TESE-ICSI embryos, however, was not significantly different from the expansion rate of IVF embryos (beta -43.7µm^2^/hour; 95% CI: -113.5 to 26.1) (Table 2, Model 1b).

Additionally, we analysed if the timing of tB was different between the three fertilization method groups, as this could impact on the time window between tB and embryo selection, and therefore the time during which expansion could occur. In ICSI and TESE-ICSI embryos, tB occurred significantly earlier than in IVF embryos (beta -2.0 h; 95% CI: -3.7 to -0.4 and -1.6 h; 95% CI: -3.2 to -0.01, respectively). However, ICSI embryos have been described to have an earlier starting point than IVF embryos, as fertilization is initiated directly after ICSI. We therefore also calculated tB from the time of pronuclear fading (tB-tPNf), after which no significant differences remained between ICSI and IVF embryos for reaching the full blastocyst stage (data not shown).

### Blastocoel size and expansion rate of transferred embryos that did or did not successfully implant

Baseline characteristics of all cycles with fresh SET, and in the case of DET, if transfer resulted in no implantation or implantation of both embryos, are shown (Additional file 2). No significant differences were found in the blastocoel surface measurements or expansion rate between embryos resulting in a biochemical pregnancy and embryos that did not result in a biochemical pregnancy (Additional file 3). However, the longitudinal blastocoel surface measurements of embryos resulting in an ongoing pregnancy showed a significantly increased size (beta 795.4 µm^2^; 95% CI: 15.4 to 1575.4) compared with the blastocoel of embryos that did not result in an ongoing pregnancy (Table 3, Model 2a). This means that the blastocoel size of embryos resulting in an ongoing pregnancy was on average 795.4 µm^2^ larger during the entire expansion trajectory than the blastocoel of embryos that did not result in an ongoing pregnancy. This is illustrated with blastocyst expansion trend lines of embryos leading to an ongoing pregnancy versus no ongoing pregnancy (Fig. 3b). Starting from 6 h post tB, the blastocoel of embryos that resulted in an ongoing pregnancy becomes larger than the blastocoel of embryos that did not result in an ongoing pregnancy. A comparison of the expansion rate of each embryo between the two groups revealed that the blastocoel of embryos resulting in an ongoing pregnancy expands significantly faster (beta 100.9 µm^2^/hour; 95% CI: 5.7 to 196.2) than the blastocoel of embryos that did not result in an ongoing pregnancy (Table 3, Model 2b).

**Table 3:** Linear mixed model analysis of blastocyst expansion surface measurements over time and the expansion rate, of fresh embryo transfers (SET and DET resulting in either no ongoing pregnancy or a twin ongoing pregnancy) compared between no ongoing pregnancy and ongoing pregnancy

	Model 1a Beta [95% CI] µm^2^			Model 2a Beta [95% CI] µm^2^		
	Ongoing pregnancy	*p*-value	No ongoing pregnancy	Ongoing pregnancy	*p*-value	No ongoing pregnancy
Surface	829.3 [58.0 to 1600.5]	0.035	ref	795.4 [15.4 to 1575.4]	0.046	ref
	Model 1b Beta [95% CI] µm^2^/hour			Model 2b Beta [95% CI] µm^2^/hour		
Expansion rate	109.2 [15.0 to 203.6]	0.023	ref	100.9 [5.7 to 196.2]	0.038	ref

Beta’s are reported as estimates in µm^2^ or µm^2^/hour. Model 1a: adjusted for tB; Model 1b: crude; Model 2a: tB and female age; Model 2b: adjusted for female age. A *p*-value of < 0.05 was considered significant.

*Abbreviations:* *tB* time to full Blastocyst, *ref* reference

We also investigated if differences exist in blastocoel size and expansion rate within the group of transferred embryos between different fertilization methods. Linear mixed model analysis showed that the blastocoel of transferred ICSI and TESE-ICSI blastocysts were overall smaller (beta -1237.8 µm; 95% CI: -2168.6 to -306.9; beta -871.7; 95% CI: -1748.6 to 5.2, respectively) than the blastocoel of IVF blastocysts (Additional file 5, model 2a). However, blastocysts resulting from the three fertilization methods showed similar expansion rates (Additional file 5, model 1b and 2b).

## Discussion

Our study quantitatively followed the dynamics of blastocoel expansion over time. We found that the blastocoel of ICSI and TESE-ICSI embryos is overall smaller than the blastocoel of IVF embryos. In addition, the expansion rate of ICSI with ejaculated sperm embryos was lower than the expansion rate of both IVF and TESE-ICSI embryos. Interestingly, we found that a larger size of the blastocoel and faster expansion were associated with ongoing pregnancy.

The observed smaller size and lower expansion rate for embryos that did not result in an ongoing pregnancy might have a genetic cause. A previous study observed in a small cohort of embryos undergoing preimplantation genetic testing for aneuploidy (PGT-A) that euploid blastocysts expanded significantly faster than aneuploid blastocysts [25]. Another study compared mitotic spindles in slow and fast developing human blastocysts. They observed that slow developing blastocysts more frequently have abnormal spindles that could lead to cellular mitotic arrest and consequently reduced cell numbers [26]. Moreover, in a mouse model for mosaicism in which mouse chimeras were created by using a mixture of normal and chemically induced aneuploid cells, it was shown that aneuploid cells showed proliferation defects in the TE, whereas they were actively eliminated by apoptosis in the ICM [27]. Thus, aneuploidy may result in fewer TE cells, negatively impacting blastocyst expansion on day 5. In line with this, a positive correlation has been described between expansion rate and the number of TE cells in human frozen-thawed surplus blastocysts, where hatched blastocysts had a higher number of TE cells [28]. On the other hand, research in mouse embryos has also shown an important role for the ICM in the proliferation of the TE, as the ICM produces the signaling factor FGF4, which regulates TE development [29–31]. Emerging research from mouse blastoids, a blastocyst model generated from embryonic and trophoblast stem cells, further demonstrated that the reconstituted ICM functionally regulated the diameter of the blastocoel by producing specific signaling factors [32]. Thus, blastocoel expansion could also be a readout of the developmental progression of the ICM.

The association between the dynamics of blastocoel size and the different fertilization methods can give more insight into the biological consequences of performing ICSI. Our findings of a smaller blastocoel of ICSI and TESE-ICSI embryos is in line with an earlier observation of a smaller diameter of frozen-thawed ICSI blastocysts at hatching commencement than IVF blastocysts [33]. Also, a lower complete hatching rate was shown in ICSI embryos than in IVF embryos, suggesting that there was insufficient expansion in the ICSI group [33]. This study observed a small slit in the zona pellucida (ZP) in some ICSI-derived blastocysts that resulted in the herniation of some TE cells. The difference in expansion rate could thus have a mechanical cause, as the introduction of the injection pipette through the ZP results in a small hole. This might result in a smaller maximum expansion size for ICSI and TESE-ICSI embryos. Interestingly, the expansion rate of TESE-ICSI embryos was faster than the expansion rate of ICSI embryos. For this finding, we do not have a clear explanation. We hypothesize that it could be related to oocyte quality. A previous study has shown that cleavage stage morphokinetics and the time of blastulation were not affected by male factor infertility after controlling for female factors [34]. These findings suggest that, at least until the start of blastulation, the embryo developmental dynamics are mainly controlled by the oocyte and factors impacting oocyte quality [34]. This effect may thus continue after blastulation. Fertilization rates in our cohort were lower after TESE-ICSI and this might give rise to a selection of only good quality oocytes that can sustain fertilization by testicular spermatozoa.

**Fig. 4:**
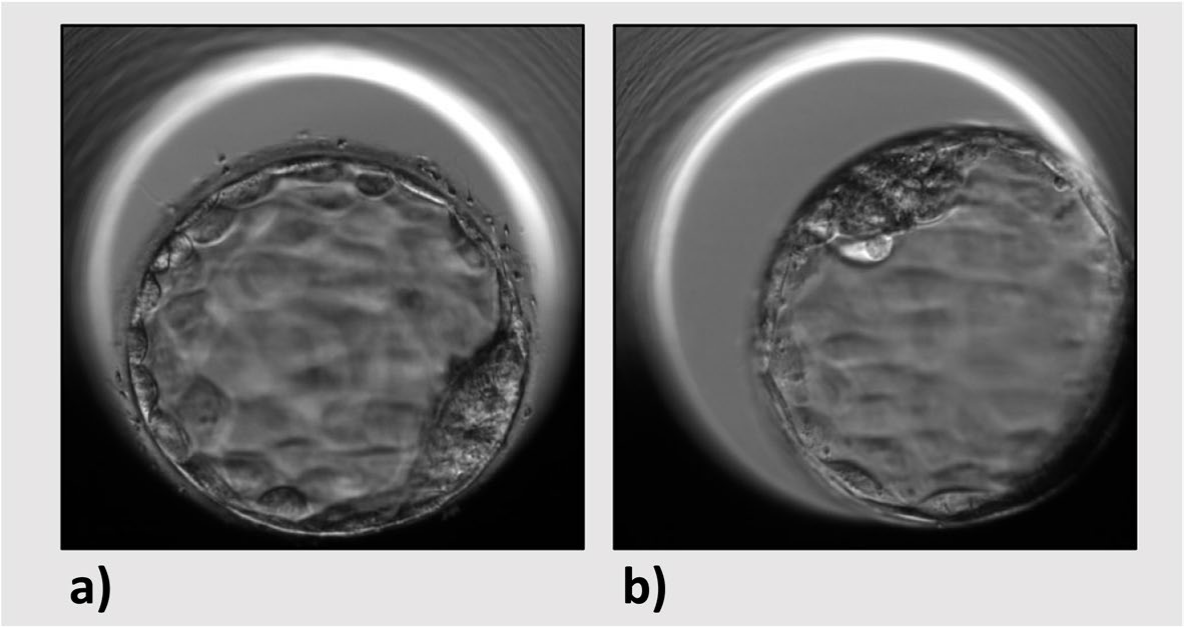
EmbryoScope images of two blastocysts from the same patient with similar morphology (B411) at 116 h post-fertilization, but different expansion rates. **a** Blastocyst with an expansion rate of 1486 µm^2^/hour. **b** Blastocyst with an expansion rate of 957 µm^2^/hour

The stage of blastocyst development is already an important parameter for embryo selection on day 5, known to be associated with clinical pregnancy and live birth [14, 16, 35–37]. One previous study performed static measurements of blastocyst size before embryo transfer. They showed that blastocysts resulting in a clinical pregnancy were significantly larger than those that did not result in a clinical pregnancy [38]. An advantage of our dynamic measurements is that we were able to calculate the expansion rate. Our results indicate that the blastocoel expansion rate has a stronger association with ongoing pregnancy than the blastocoel surface measurements over time. Within the transferred embryo group, we observed ICSI embryos to be smaller than IVF embryos, but the expansion rate was similar. The expansion rate can thus be a valuable parameter in the embryo selection process that is independent of fertilization method. In the case of two blastocysts with similar morphology, the blastocyst expansion rate could be used to differentiate between the two (Fig. 4). In addition, the dynamics of blastocyst expansion might improve the selection of embryos that lead not just to implantation, but to an ongoing pregnancy. In daily practice, the limiting factor is that these manual measurements are time-consuming. However, surface recognition can be automated and automatic blastocoel surface measurements might thus be easily incorporated in embryo selection algorithms. In this study, calculation of the expansion rate was made easily applicable for daily clinical practice, by determining the maximal expansion surface and subtracting the minimum surface at tB, divided by the number of hours needed to reach the maximum expansion. However, blastocysts are also known to sometimes collapse the blastocoel during development, with a potential impact on implantation potential [39–41]. The expansion rate we calculated here is independent of these collapses, because we used the maximum size that each blastocoel reached. For future research, it would be interesting to incorporate the number and extent of blastocoel collapses into such analysis, to further refine the model.

A strength of our study is that we used dynamic measurements of the expanding blastocyst of a large cohort of blastocyst transfers with known pregnancy outcomes. Also, the use of linear mixed model analysis took clustering of embryos originating from the same couple into account, preventing a possible bias of a large number of embryos originating from one couple. Our results are independent of the timing of full blastocyst formation because we corrected for this in our model. However, we do acknowledge the limitation of the retrospective and observational nature of this study. All TLM studies are subject to selection bias because of the analysis of fresh embryo transfer with the treatment outcome. These embryos, and the transferred embryos in our study, are already chosen as the best embryos by morphological criteria, where the blastocyst expansion stage is an important selection criterion. Therefore, prospective validation of our results should be performed before application into clinical practice.

## Conclusion

The blastocoel of both ICSI embryos and TESE-ICSI embryos was significantly smaller than the blastocoel of IVF embryos. Still, the blastocoel of transferred embryos resulting in an ongoing pregnancy was significantly larger and expanded significantly faster than the blastocoel of transferred embryos that did not, regardless of the fertilization method. Longitudinal blastocyst surface measurements and expansion rates are potential non-invasive quantitative markers that can aid in the embryo selection process. Future research would be to automate these measurements and to investigate the predictive value of adding these parameters to already existing TLM prediction models.

## Supplementary Information

**Additional file 1.** Baselinecharacteristics and treatment outcomes of excluded cycles (cycleswithout embryos that reached the fullblastocyst stage).

**Additional file 2.** Baselinecharacteristics of cycles of fresh embryo transfers (SET and DETresulting in either no ongoing pregnancy or atwin ongoing pregnancy).

**Additional file 3.** Linearmixed model analysis of blastocyst expansion surface measurementsover time and the expansion rate, of freshembryo transfers (SET and DET resulting in either noimplantationor implantation of both embryos) compared between no pregnancy and biochemicalpregnancy.

**Additional file 4.** Linear mixed model analysis of blastocystexpansion surface measurements over time compared between different ovarianstimulation protocols and different culture media.

**Additional file 5.** Linear mixed model analysis of blastocystexpansion surface measurements over time and expansion rate, of fresh embryotransfers (SET and DET resulting in either no ongoing pregnancy or a twinongoing pregnancy) compared between IVF, ICSI with ejaculated sperm andTESE-ICSI.

## Data Availability

The data underlying this article cannot be shared publicly due to the privacy of individuals that participated in the study. The data will be shared on reasonable request to the corresponding author.

## Abbreviations

IVF: In vitro Fertilization
ICSI: Intracytoplasmic sperm injection
TESE-ICS: Testicular sperm extraction combined with intracytoplasmic sperm injection
tB: Time of full blastocyst formation
CI: Confidence interval
SET: Single embryo transfer
TE: Trophectoderm
ICM: Inner cell mass
TLM: Time-lapse morphokinetic
DET: Double embryo transfer
GnRH: Gonadotropin-releasing hormone
hCG: Human chorionic gonadotropin
tPNf: Time of pronuclear fading
ZP: Zona pellucida
