# Supplementary material for: Longitudinal surface measurements of human blastocysts show that the dynamics of blastocoel expansion are associated with fertilization method and ongoing pregnancy
Source: Reprod Biol Endocrinol. 2022 Mar 19;20:53. doi: 10.1186/s12958-022-00917-2 (PMC8933899; doi:10.1186/s12958-022-00917-2)
Supplement: Supplementary file 1 — Additional file 1. Baselinecharacteristics and treatment outcomes of excluded cycles (cycleswithout embryos that reached the fullblastocyst stage). [file 12958_2022_917_MOESM1_ESM.docx]

**Additional file 1** Baseline characteristics and treatment outcomes of excluded cycles (cycles without embryos that reached the full blastocyst stage)

|  | **Excluded cycles**  (n= 18) |
| --- | --- |
| **Fertilization method** |  |
| IVF | 5 (27.8%)) |
| ICSI with ejaculated sperm | 3 (16.7%) |
| TESE-ICSI | 10 (55.6%) |
| **Female age** | 34.7 (30.4-38.3) |
| **Male age** | 36.5 (32.0-42.3) |
| **Oocytes aspirated** | 9 (4-11) |
| **Stimulation Protocol** |  |
| GnRH-antagonist | 7 (38.9%) |
| GnRH-agonist | 11 (61.1%) |
| **Culture medium** |  |
| Sage1 | 10 (55.6%) |
| Vitrolife G-TL | 8 (44.4%) |
| **Embryo transfer** |  |
| 0 | 1 (5.6%) |
| 1 | 16 (88.9%( |
| 2 | 1 (5.6%) |
| **Biochemical pregnancy** | 2 (11.8%) |
| **Ongoing pregnancy** | 0 (0%) |

Each cycle is derived from a unique patient couple. Data are presented as number (%) or median (interquartile range). Abbreviations: IVF, *in vitro* fertilization; ICSI, intracytoplasmic sperm injection; TESE-ICSI, testicular sperm extraction with intracytoplasmic sperm injection; GnRH, gonadotropin-releasing hormone.
