# Supplementary material for: Longitudinal surface measurements of human blastocysts show that the dynamics of blastocoel expansion are associated with fertilization method and ongoing pregnancy
Source: Reprod Biol Endocrinol. 2022 Mar 19;20:53. doi: 10.1186/s12958-022-00917-2 (PMC8933899; doi:10.1186/s12958-022-00917-2)
Supplement: Supplementary file 2 — Additional file 2. Baselinecharacteristics of cycles of fresh embryo transfers (SET and DETresulting in either no ongoing pregnancy or atwin ongoing pregnancy). [file 12958_2022_917_MOESM2_ESM.docx]

**Additional file 2** Baseline characteristics of cycles of fresh embryo transfers (SET and DET resulting in either no ongoing pregnancy or a twin ongoing pregnancy)

|  | **No ongoing pregnancy**  (n=125 ) | **Ongoing pregnancy**  (n=68 ) | **p-value** |
| --- | --- | --- | --- |
| **Fertilization method** |  |  | 0.580 |
| IVF | 42 (33.6%) | 26 (38.2%) |  |
| ICSI with ejaculated sperm | 35 (28.0%) | 21 (30.9%) |  |
| TESE-ICSI | 48 (38.4%) | 21 (30.9%) |  |
| **Female age** | 34.9 (31.7-38.3) | 33.4 (30.1-36.5) | 0.057 |
| **Male age** | 36.0 (32.5-40.0) | 35.0 (32.0-39.0) | 0.637 |
| **Oocytes aspirated** | 8 (6-12) | 10 (7-12) | 0.560 |
| **Stimulation Protocol** |  |  | 0.541 |
| GnRH-antagonist | 81 (64.8%) | 46 (67.6%) |  |
| GnRH-agonist | 39 (31.2%) | 18 (26.5%) |  |
| missing | 5 (4.0%) | 4 (5.9%) |  |
| **Culture medium** |  |  | 0.251 |
| Sage1 | 49 (39.2%) | 21 (30.9%) |  |
| Vitrolife G-TL | 76 (60.8%) | 47 (69.1%) |  |

Each cycle is derived from a unique patient couple. Data are presented as number (%) or median (interquartile range). A p-value of <0.05 was considered significant. Abbreviations: IVF, *in vitro* fertilization; ICSI, intracytoplasmic sperm injection; TESE-ICSI, testicular sperm extraction with intracytoplasmic sperm injection; GnRH, gonadotropin-releasing hormone.
