# Supplementary material for: Longitudinal surface measurements of human blastocysts show that the dynamics of blastocoel expansion are associated with fertilization method and ongoing pregnancy
Source: Reprod Biol Endocrinol. 2022 Mar 19;20:53. doi: 10.1186/s12958-022-00917-2 (PMC8933899; doi:10.1186/s12958-022-00917-2)
Supplement: Supplementary file 3 — Additional file 3. Linearmixed model analysis of blastocyst expansion surface measurementsover time and the expansion rate, of freshembryo transfers (SET and DET resulting in either noimplantationor implantation of both embryos) compared between no pregnancy and biochemicalpregnancy. [file 12958_2022_917_MOESM3_ESM.docx]

**Additional file 3** Linear mixed model analysis of blastocyst expansion surface measurements over time and the expansion rate, of fresh embryo transfers (SET and DET resulting in either no implantation or implantation of both embryos) compared between no biochemical pregnancy and biochemical pregnancy

|  | **Model 1a**  **Beta [95% CI]**  **µm^2^** | | | **Model 2a**  **Beta [95% CI]**  **µm^2^** | | |
| --- | --- | --- | --- | --- | --- | --- |
|  | **Biochemical pregnancy** | **p-value** | **No pregnancy** | **Biochemical pregnancy** | **p-value** | **No pregnancy** |
| **Surface** | 622.6  [-133.2 to 1378.5] | 0.106 | ref | 578.3  [-192.5 to 1349.1] | 0.141 | ref |
|  | **Model 1b**  **Beta [95% CI]**  **µm^2^/hour** | | | **Model 2b**  **Beta [95% CI]**  **µm^2^** | | |
| **Expansion rate** | 88.0  [-2.86 to 178.9] | 0.058 | ref | 77.6  [-15.1 to 170.2] | 0.100 | ref |

Beta’s are reported as estimates in µm^2^ or µm^2^/hour. Model 1a: adjusted for tB; Model 1b: crude; Model 2a: tB and female age; Model 2b: adjusted for female age. A p-value of <0.05 was considered significant. Abbreviations: tB, time to full blastocyst; ref, reference.
