# Supplementary material for: Longitudinal surface measurements of human blastocysts show that the dynamics of blastocoel expansion are associated with fertilization method and ongoing pregnancy
Source: Reprod Biol Endocrinol. 2022 Mar 19;20:53. doi: 10.1186/s12958-022-00917-2 (PMC8933899; doi:10.1186/s12958-022-00917-2)
Supplement: Supplementary file 4 — Additional file 4. Linear mixed model analysis of blastocystexpansion surface measurements over time compared between different ovarianstimulation protocols and different culture media. [file 12958_2022_917_MOESM4_ESM.docx]

**Additional file 4** Linear mixed model analysis of blastocyst expansion surface measurements over time compared between different ovarian stimulation protocols and different culture media

|  | **Model 1**  **Beta [95% CI]**  **µm^2^** | | | **Model 2**  **Beta [95% CI]**  **µm^2^** | | |
| --- | --- | --- | --- | --- | --- | --- |
|  | **GnRH-antagonist** | **p-value** | **GnRH-agonist** | **Sage 1** | **p-value** | **Vitrolife G-TL** |
| **Surface** | -336.3 [-784.6 to 112.1] | 0.141 | ref | 269.6 [-159.2 to 698.4] | 0.217 | ref |

Beta’s are reported as estimates in µm^2^. Model 1: Comparison of blastocyst surface between embryos resulting from a GnRH-antagonist or –agonist ovarian stimulation protocol, adjusted for tB; Model 2: Comparison of blastocyst surface between embryos cultured in Sage 1 (Origio/Cooper Surgical, Trumbull, CT, USA) or Vitrolife G-TL (Vitrolife, Göteborg, Sweden) culture medium, adjusted for tB. A p-value of <0.05 was considered significant. Abbreviations: tB, time to full blastocyst.
