# Supplementary material for: Longitudinal surface measurements of human blastocysts show that the dynamics of blastocoel expansion are associated with fertilization method and ongoing pregnancy
Source: Reprod Biol Endocrinol. 2022 Mar 19;20:53. doi: 10.1186/s12958-022-00917-2 (PMC8933899; doi:10.1186/s12958-022-00917-2)
Supplement: Supplementary file 5 — Additional file 5. Linear mixed model analysis of blastocystexpansion surface measurements over time and expansion rate, of fresh embryotransfers (SET and DET resulting in either no ongoing pregnancy or a twinongoing pregnancy) compared between IVF, ICSI with ejaculated sperm andTESE-ICSI. [file 12958_2022_917_MOESM5_ESM.docx]

**Additional file 5** Linear mixed model analysis of blastocyst expansion surface measurements over time and expansion rate, of fresh embryo transfers (SET and DET resulting in either no ongoing pregnancy or a twin ongoing pregnancy) compared between IVF, ICSI with ejaculated sperm and TESE-ICSI

|  | **Model 1a**  **Beta [95% CI]**  **µm^2^** | | | | | **Model 2a**  **Beta [95% CI]**  **µm^2^** | | | | |
| --- | --- | --- | --- | --- | --- | --- | --- | --- | --- | --- |
|  | **TESE-ICSI** | **p-value** | **ICSI**  **(ejaculated sperm)** | **p-value** | **IVF** | **TESE-ICSI** | **p-value** | **ICSI (ejaculated sperm)** | **p-value** | **IVF** |
| **Surface** | -826.7  [-1702.2 to 48.9] | 0.064 | -1154.9  [-2078.4 to -231.4] | 0.015 | ref | -871.7  [-1748.6 to 5.2] | 0.051 | -1237.8  [-2168.6 to -306.9] | 0.009 | ref |
|  | **Model 1b**  **Beta [95% CI]**  **µm^2^/hour** | | | | | **Model 2b**  **Beta [95% CI]**  **µm^2^/hour** | | | | |
| **Expansion rate** | -72.6  [-181.2 to 35.9] | 0.188 | -82.7  [-196.8 to -31.4] | 0.155 | ref | -83.0  [-191.4 to 25.4] | 0.132 | -101.4  [-216.6 to 13.7] | 0.084 | ref |

Beta’s are reported as estimates in µm^2^. Model 1a: adjusted for tB; Model 1b: crude; Model 2a: tB and female age; Model 2b: adjusted for female age. A p-value of <0.05 was considered significant. Abbreviations: tB, time to full blastocyst.
